# Chromosomal evolution of rDNA and H3 histone genes in representative Romaleidae grasshoppers from northeast Brazil

**DOI:** 10.1186/1755-8166-6-41

**Published:** 2013-10-03

**Authors:** Marcos S Regueira Neto, Maria José de Souza, Vilma Loreto

**Affiliations:** 1 Departamento de Genética, CCB, Universidade Federal de Pernambuco, Pernambuco, Recife, Brazil

**Keywords:** Chromosome, Fluorescent in situ hybridization, H3, rDNA

## Abstract

**Background:**

Grasshoppers from the Romaleidae family are well distributed in the Neotropical Region and represent a diversified and multicolored group in which the karyotype is conserved. Few studies have been conducted to understand the evolutionary dynamics of multigene families. Here, we report the chromosomal locations of the 18S and 5S rDNA and H3 histone multigene families in four grasshopper species from the Romaleidae family, revealed by fluorescent in situ hybridization (FISH).

**Results:**

The 5S rDNA gene was located in one or two chromosome pairs, depending on the species, and was found in a basal distribution pattern. Its chromosomal location was highly conserved among these species. The 18S rDNA was located in a single medium-sized chromosomal pair in all species analyzed. Its chromosomal location was near the centromere in the proximal or pericentromeric regions. The location of the H3 histone gene was highly conserved, with slight chromosomal location differences among some species. To our knowledge, this is the first report of a megameric chromosome carrying both the chromosomal markers 18S rDNA and the H3 histone genes, thereby expanding our understanding of such chromosomes.

**Conclusions:**

The 5S and 18S rDNA genes and the H3 histone genes showed a conservative pattern in the species that we analyzed. A basal distribution pattern for 5S rDNA was observed with a location on the fourth chromosomal pair, and it was identified as the possible ancestral bearer. The 18S rDNA and H3 histone genes were restricted to a single pair of chromosomes, representing an ancestral pattern. Our results reinforce the known taxonomic relationships between *Chromacris* and *Xestotrachelus*, which are two close genera.

## Background

Repetitive DNA elements constitute a large portion of eukaryotic genomes. These elements include tandemly arrayed satellites, minisatellites and microsatellites, multigene families, and dispersed repeats, such as transposons and retrotransposons [1]. The multigene families (hemoglobins, actins, histones, and ribosomal RNAs) are composed of genes with the same functions, with a structure and origin from a common ancestral gene, and it is assumed that multigene family members have undergone concerted evolution [2,3]. In higher eukaryotes, the ribosomal RNA genes are tandemly arrayed and organized in multigene families. The major ribosomal DNA family is formed by the association of one copy of 28S, 5,8S, and 18S rRNA, and these genes are separated by internal transcribed spacers (ITS1 and ITS2).

Another ribosomal RNA gene is 5S rRNA, with a length of 160 bp. The repeats of this gene are separated by non-transcribed spacers (NTS). Although the 45S rRNA subunits are synthesized, processed, and partially assembled to form the ribosome subunits in the nucleolus, the 5S rRNA is synthesized elsewhere in the genome [1,4].

The histone genes can be organized in one cluster formed by all four core histones (H2A, H2B, H3, and H4) or a quintet (H1, H2A, H2B, H3, and H4), and the repeats are tandemly arrayed and spaced by non-transcribed intergenic spacers (IGS) [3,5,6].

These two multigene families (rDNA genes and histone) are excellent chromosomal markers for understanding karyotype evolution and genome organization in many groups, due to their clustered organization. rRNA genes have already been mapped in many groups of invertebrates, such as worms, insects, mollusks, and echinoderms, using fluorescent in situ hybridization [7-11], and variations have been found among these different organisms. The histone gene has been mapped in amphibians, insects, mammals, and mollusks. In contrast with rRNA genes, it is conservative in its chromosome location and number of clusters [11-14].

The distributions of rDNA and histone genes have been studied in some groups of grasshoppers, such as the Acrididae, Ommexechidae, and Proscopiidae families [10,15,16]. Different groups show large variation in the location of ribosomal genes. In some species, the genes are located in only one chromosome pair, and in others they are located in almost all the complementary chromosomes. In grasshoppers, the 45S and 5S rRNA genes are most commonly located in different chromosomes, but both genes can also occur in colocalized positions [10,17]. The histone genes have also been mapped in grasshoppers from the Acrididae and Proscopiidae families. They show a conserved pattern of gene clusters with respect to their numbers and chromosomal location. There was an observed association among the 5S rRNA and H3 histone genes [16,18].

The distribution of Romaleidae grasshoppers is predominant in the Neotropical Region, despite the invasion of the Nearctic Region by some genera. In northeast Brazil, they occur from the Atlantic Forest to Caatinga. More than 80 genera of grasshoppers have been taxonomically described within the Romaleidae family [19]. That group shows a predominance of conservative karyotypes and the majority of species have a diploid number 2n = 23,X0 in males and 2n = 24,XX in females, with acrocentric chromosomes [20]. Fluorescent in situ hybridization (FISH) data are scarce; a few species have been studied in Romaleidae, but there are no data regarding 5S rDNA and H3 histone genes in this group. The 45S rDNA was mapped using FISH in four Romaleidae species: *Chromacris nuptialis*, *C. speciosa*, *Xestotrachelus robustus*, and *Xyleus discoideus angulatus*[21-23].

In this study, we performed a comparative cytogenetic analysis of the chromosome location of three multigene families (the 5S and 18S rDNA and H3 histone genes) in four grasshopper species from the Romaleidae family by conducting simple and double FISH in order to contribute to our understanding of the evolutionary dynamics of these sequences in Orthoptera.

## Results

The diploid number in the four species studied is 2n = 23,X0 in males and 2n = 24,XX in females. All the species showed acrocentric chromosomes; in addition, *Xestotrachelus robustus* showed two meta-submetacentric chromosome pairs. *Brasilacris gigas* showed four large chromosomes (L1–L4), five medium chromosomes (M5–M9), and two small chromosomes (S10–S11), and the X chromosome was a large one. The karyotype of the two species of *Chromacris* consists of two large chromosomes (L1–L2), six medium chromosomes (M3–M8), and three small chromosomes (S9–S11). The X chromosome was medium sized. *Xestotrachelus robustus* contains three large acrocentric chromosomes (L1–L3), five medium acrocentric chromosomes (M4–M8), two small meta-submetacentric chromosomes (S9–S10), and one small acrocentric chromosome (S11). The X chromosome is the fourth in decreasing size.

**Figure 1:**
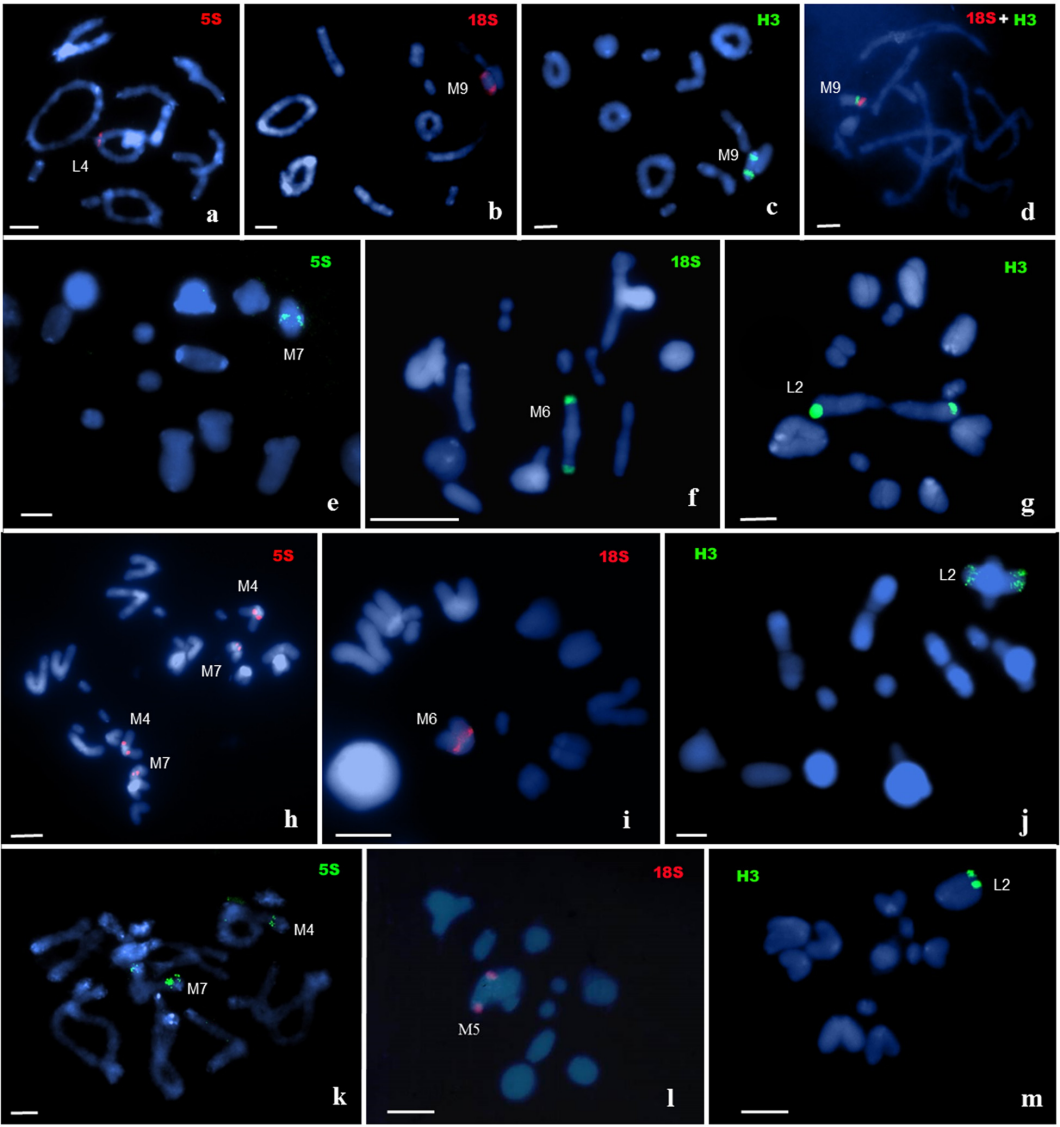
**Fluorescent in situ hybridization using the 5S rDNA (a, e, h and k), 18S rDNA (b, f, i and l) and H3 histone genes (c, g, j and m) probes in meiotic cells. (a**-**d)***Brasilacris gigas*, **(e**-**g)***Chromacris nuptialis*, **(h**-**j)***Chromacris speciosa* and **(k**, **m)***Xestotrachelus robustus*. Double FISH performed using a combination of 18S rDNA and H3 histone gene probes in *B. gigas***(d)**. Meiotic stages: pachytene **(d)**, diplotene **(a**,**b**,**c** and **k)**, metaphase I **(c**, **f**, **j** and **l)**, anaphase I **(h)** and metaphase II **(g**, **I** and **m)**. Scale bar = 5 μm.

**Figure 2:**
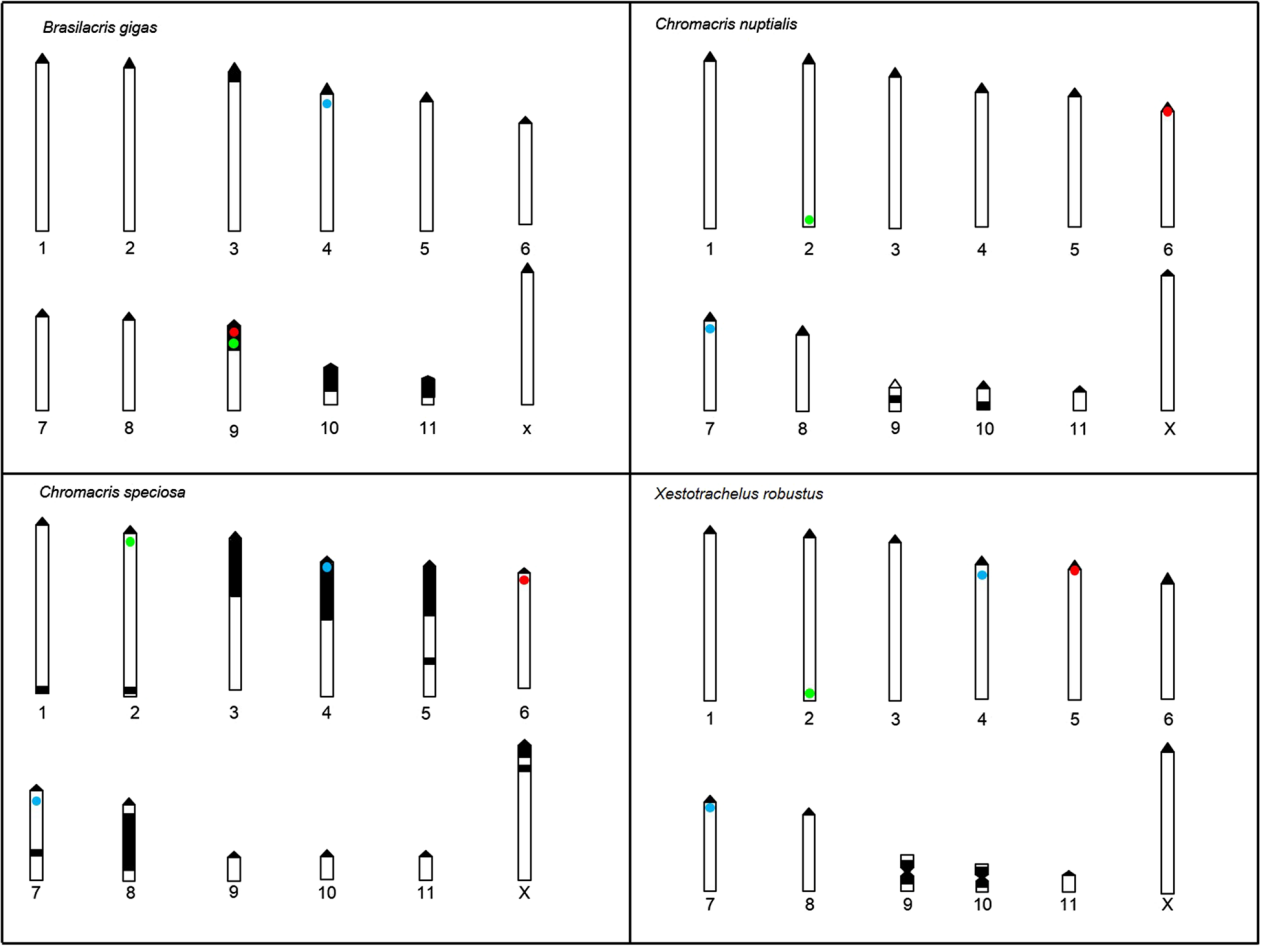
**Idiogram showing the chromosomal location of 5S rDNA, 18S rDNA, H3 histone genes and constitutive heterochromatin distribution pattern in the four Romaleidae grasshoppers.** The black, chromosome regions represent the constitutive heterochromatin [22,30] and the blue, red and green areas represent the 5S rDNA, 18S rDNA and the H3 histone genes, respectively.

In *B. gigas*, the 5S rDNA site was present in the fourth chromosome pair (Figure 1a), and the 18S rDNA and H3 histone genes were in the M9 chromosome (Figures 1b, c, and d). In *B. gigas*, all genes are located in the proximal region (Figure 2). In *B. gigas*, we found both 18S and H3 genes in the same chromosome and position. The results of double FISH in *B. gigas* showed that the 18S rDNA and H3 histone genes were contiguous in the proximal region of the megameric M9 chromosome (Figure 1d). In *C. nuptialis*, the 5S rDNA, 18S rDNA, and H3 histone genes were present in the M7 (Figure 1e), M6 (Figure 1f), and L2 (Figure 1g) chromosomes, respectively. The 5S rDNA, 18S rDNA, and H3 histone genes were located at the proximal, pericentromeric, and terminal regions of chromosomes, respectively (Figure 2). In *C. speciosa*, the 5S rDNA was located in the M4 and M7 (Figure 1h) chromosomes, and the 18S rDNA and H3 histone genes were located in the M6 (Figure 1i) and L2 (Figure 1j) chromosomes, respectively. All genes in *C. speciosa* were located at the proximal regions of chromosomes (Figure 2). In *X. robustus*, the 5S rDNA was located in the M4 and M7 (Figure 1k) chromosomes, and the 18S rDNA and H3 histone genes were located in the M5 (Figure 1l) and L2 (Figure 1m) chromosomes, respectively. The 5S rDNA, 18S rDNA, and H3 histone genes were located at the proximal, pericentromeric, and terminal regions of chromosomes in *X. robustus*, respectively (Figure 2).

## Discussion

Two different distribution patterns for 5S rDNA sites were revealed by FISH in the four species of grasshoppers. In the first pattern, the site is restricted to a single pair of chromosomes, and in the second pattern, two sites are located in different chromosome pairs. We found six sites in all, including three located in the fourth chromosome and the three in the seventh chromosome, in the order of decreasing size. One or two chromosome pairs with the 5S rRNA gene were reported in 29 grasshopper species from the Acrididae family [24]. Of these 29 species, 13 showed multiple 5S rDNA sites located in the fourth or seventh pairs of chromosomes or even in both pairs (fourth and seventh). In addition, four representatives of the family Proscopiidae, which is composed of the most primitive grasshoppers, showed the 5S rDNA gene located only in the fourth autosomal pair [16]. The 5S rDNA in the fourth pair is also present in *B. gigas*, *C. speciosa*, and *X. robustus*, supported by the results from the Acrididae and Proscopiidae families [16,24], which suggests that this may be an ancient placement for these genes. *C. nuptialis* probably lost the site in the fourth pair in the course of evolution.

The 5S rDNA gene observed in the seventh chromosome pair may be an apomorphic condition in grasshopper species in this study, and it may be caused by specific spread mechanisms that remain unclear. Our data also shows all sites located in the proximal region of a medium-sized chromosome, suggesting that this is an ancestral condition for Romaleidae grasshoppers. Despite the spread of this sequence through the Romaleidae genome, the cluster location of the 5S rDNA gene has been restricted to this chromosomal group. We presume that the proximal location of the 5S rDNA cluster is the optimal condition in grasshoppers and probably the ancient one; the other patterns may represent a derived condition. This understanding is also supported by the proximal location of 5S rDNA clusters found in 75% of 29 species of Acrididae and three species of Proscopiidae grasshoppers analyzed [16,24].

Data on the 18S rDNA gene are scarce in the Romaleidae family, as well as in Ommexechidae and Proscopiidae. Data may be most plentiful for the Acrididae family [10,15,16,24]. The 18S rDNA clusters are restricted to a single medium-sized autosomal pair in *C. nuptialis*, *C. speciosa*, and *X. robustus*, corroborating previous studies from Loreto et al. [23] and Souza et al. [22], which suggests that it may be the ancestral bearer of 18S rDNA. This hypothesis is supported by the data from *X. d. angulatus,* another Romaleidae grasshopper, which showed the 18S rDNA gene in a medium-sized pair [21]. In a study of 47 species of Acrididae and two species of Romaleidae, Cabrero and Camacho [10] observed that in 32.7% of the species the cluster of 45S rDNA was in a single pair, in 37.7% the site was located in two chromosomes, and in 30.6% the 45S rDNA cluster was in multiple pairs of chromosomes. These data show the 45S rDNA spread through the grasshoppers’ genome in the course of evolution. Two species of Ommexechidae, *Ommexecha virens* and *Descampsacris serrulatum* had three and four 45S rDNA sites, respectively. These data show a possible spread of this cluster in these two species [15]. However, in four Proscopiidae grasshoppers there was a consensus location in the seventh chromosome pair, indicating an ancestral condition with this single pair as the original bearer of the 18S rDNA cluster and the extra sites present in two species as a derived pattern [16].

As a number of species have only a single site for 45S rDNA,, there may be some restrictions on high numbers of rDNA loci within species. The spread can be explained by chromosome rearrangements, translocations or inversions, ectopic recombination, and transposition of some rDNA sequences to new places in the same or different chromosomes [10]. In grasshoppers, the rDNA is sometimes located in constitutive heterochromatin (CH) regions, so a possible rDNA spread mechanism may be associated with CH spread. Previous studies have reported an amplification and dispersion of CH in eight species from six genera (*Brasilacris*, *Chromacris*, *Phaeoparia*, *Radacridium*, *Xestotrachelus*, and *Xyleus*) from the Romaleidae family [21,23,25-27]. On the other hand, when data from the 10 ribosomal sites found in *B. gigas*, *C. nuptialis*, *C. speciosa*, and *X. robustus* are correlated with information from previous studies [22,25] related to the CH pattern, the results indicate four ribosomal sites in CH regions and six ribosomal sites outside CH regions.

In our study, due to the single site for rDNA in the four species studied, we can infer two important aspects of the location of the nucleolus organizer regions (NORs) in this group: i) The distribution of NORs seems to be well conserved and there are few rearrangements of location and expression of these sequences, and ii) the presence of only one NOR is sufficient to provide the cellular processes in these species. In the megameric chromosome M9 from *B. gigas*, there is a conspicuous CH block [25], which encompasses both the 18S rDNA and H3 histone genes, and this arrangement may be associated with a rearrangement leading to a contiguous configuration of these genes. The difference in chromosomal location of the 18S rDNA gene between the two *Chromacris* species is probably due to a paracentric inversion, an unequal crossover, or even a heterochromatin amplification that led to this modification.

As observed in humans and plants, the rDNA multigene family does not have a random distribution in karyotypes with acrocentric chromosomes [28-30]. This condition was observed in the 5S and 18S rDNA distribution pattern in grasshoppers with acrocentric chromosomes from different families (Acrididae, Ommexechidae, Proscopiidae, and Romaleidae). This pattern found in representatives of Romaleidae might suggest that the ancestral chromosome location for this group is near the centromere. In support of this idea, studies have reported the proximal chromosomal location of active NORs in some Romaleidae grasshoppers, including *Radacridium mariajoseae*, *X. d. angulatus*, and *Phaeoparia megacephala*, [21,26,27]. In 47 Old World grasshoppers, the 126 rDNA sites detected by FISH showed that 52.4% were located in the proximal region, 34.9% were interstitial, and only 12.7% were distal [10]. The majority of sites mapped to date in grasshoppers were located in the proximal or pericentromeric regions in acrocentric chromosomes [10,15,16].

In the four species of Romaleidae analyzed in our study, we did not observe an association between the two 5S and 18S rDNA markers. They were located in different chromosome pairs. This finding suggests that there are separate evolutionary pathways for these two multigene families in the Romaleidae grasshoppers. This condition is predominant among eukaryotes and was also observed in other grasshoppers from the Acrididae and Proscopiidae families. However, extreme cases have been reported in one grasshopper species *Omocestus bolivari*, which carries both rDNA types in all its chromosomes [16,24].

The H3 histone gene has been mapped in 35 representatives from Acrididae and four representatives from Proscopiidae. In all cases, the H3 histone gene cluster was restricted to a single autosomal pair [16,18]. Herein, the H3 histone gene distribution pattern is described for the first time for Romaleidae grasshoppers. We found that the location of the H3 gene was highly conservative in the four species, with slight modifications concerning location within the chromosome. In all species, the H3 histone gene site is restricted to a single autosome pair. For *Chromacris* species and *X. robustus*, the H3 cluster was located in the L2 chromosome, and for *B. gigas*, it was in the megameric M9. The chromosomal location of the H3 gene cluster was proximal in *B. gigas* and *C. speciosa* and terminal in *C. nuptialis* and *X. robustus*. The differences in the location of the H3 gene between the two *Chromacris* species are probably due to chromosomal rearrangements or transposition.

Due to morphologic similarities like wing patterns and phallic structures between the genera *Chromacris* and *Xestotrachelus*, Roberts and Carbonell [31] report in a revision that these two genera are close. Our cytogenetic study showed a similar pattern among the species from the two genera and corroborates the taxonomic relationships among them.

Double FISH revealed the 18S rDNA and H3 histone genes in the same megameric chromosome in *B. gigas*, indicating a contiguous arrangement of these two genes in the proximal region of the M9 chromosome. According to a study of active NORs by Rufas et al. [32], megameric and sexual chromosomes are frequently involved in nucleolus organization. Megameric chromosomes show consistent positive heteropycnosis at first prophase in meiosis in orthopteran insects. Rufas et al. showed that a high proportion of Acrididae species carry active NORs in the sex and megameric chromosomes. However, no megameric chromosomes were known to carry H3 histone genes until now. Nine species of Acrididae grasshoppers showed one chromosome pair carrying rDNA-H3 histone genes, but none of the sites was present in the megameric chromosomes [10,18].

The H3 histone gene pattern found in *B. gigas* is very different from that of other Romaleidae grasshoppers. This difference is probably due to a modification of the site location from its original place in the L2 to the M9 chromosome. If there is some evolutionary mechanism that combines repetitive elements of rDNA and histone in a megameric chromosome, we do not know what it is. Further chromosomal studies must be done exploring the mobility of rDNA and the highly conservative nature of the H3 histone gene.

## Conclusions

In summary, the 5S and 18S rDNA genes were preferentially located in medium-sized chromosomes among the Romaleidae grasshoppers, showing a conservative distribution pattern for these sequences. In comparison with Proscopiidae grasshoppers [16], our study revealed a basal distribution pattern of 5S rDNA; however, further studies are required to clarify these evolutionary relationships among grasshopper families. The H3 histone gene cluster seems to be highly conservative as a cytogenetic marker in Romaleidae grasshoppers, as in other grasshoppers. These results reinforce the known taxonomic relationships between the *Chromacris* and *Xestotrachelus* genera with regard to the distribution of rDNA and H3 clusters. The mechanism that involves the megameric chromosomes, when they exist in the karyotype, as the preferential bearers for rDNA sequences remains unclear. Further studies are needed to understand this situation and the association of rDNA and histone genes in megameric chromosomes.

## Methods

A total of 31 adult males from four species of Romaleidae grasshoppers, *B. gigas*, *C. nuptialis*, *C. speciosa*, and *X. robustus*, were collected in Belo Jardim, Tabocas, Camocim de São Félix, and Buíque, respectively, all in the state of Pernambuco (Northeast Brazil). The testes of the specimens were fixed in Carnoy (3:1 ethanol:acetic acid), and the chromosome preparations were made by squashing the sample in a drop of 45% acetic acid between slide and coverslip, immersing it in liquid nitrogen, and subsequently removing the coverslip.

The DNA probes of the 5S rRNA, 18S rRNA, and H3 histone genes were obtained and labeled using polymerase chain reaction (PCR). The probes were obtained from each species’ genomic DNA. To amplify the genes of interest, we used the primers 5SrDNA F(5′-AAC GAC CAT ACC ACG CTG AA-3′) and R (5′-AAG CGG TCC CCC ATC TAA GT-3′) for the 5S rDNA gene, Sca18S1F (F 5′-CCC CGT AAT CGG AAT GAG TA-3′) and Sca18S1R (R 5′-GAG GTT TCC CGT GTT GAG TC-3′) for the 18S rDNA gene, H3F-1 (5′-ATA TCC TTR GGC ATR ATR GTG AC-3′) and H3R-1 (ATG GCT CGT ACC AAG CAG ACV GC-3′) for the H3 histone gene. The amplification of the 5S rDNA, 18S rDNA, and H3 histone genes yielded three specific bands with lengths of 160 bp, 320 bp, and 370 bp, respectively. PCR products were visualized in a 1% agarose gel. The rRNA probes were labeled with biotin-16-dUTP (Roche, Mannheim, Germany), and the probe for the H3 histone gene was labeled with digoxigenin-11-dUTP (Roche, Mannheim, Germany). FISH was performed according to Moscone et al. [33] with some modifications. To map the two multigene families (18S rRNA and H3 histone) in the same cell, double FISH procedures were performed. The preparations were dehydrated in an alcohol series (70–96%, 5 min each) and incubated in RNAse A and pepsin. The probes were mixed in a hybridization solution containing 20× SSC, formamide, dextran sulfate, and salmon sperm and were subsequently denatured. The chromosomal preparation was denatured with the hybridization mix and the renaturation was performed at 37°C overnight.

A stringency level of 72% was determined after three washes in 2× SSC at 42°C, two in 0.1× SSC at 42°C, and one in 2× SSC at room temperature (5 min each). The rDNA probes from the species *B. gigas* and *C. speciosa* and the 18S rDNA from *X. robustus* were detected with a mouse-antibiotin antibody (Dako, Denmark) and the signal was amplified using rabbit anti-mouse-TRITC (Dako, Denmark) conjugate. The 5S rDNA from the species *C. nuptialis* and *X. robustus* and the 18S rDNA from *C. nuptialis* were detected with Avidin-FITC (fluorescein isothiocyanate) (Sigma-Aldrich, St. Louis, MO, USA), and the signal was amplified with Avidin-Biotin-FITC (Sigma-Aldrich, St. Louis, MO, USA). The H3 histone probe from the four species in this work was detected with a sheep anti-digoxigenin fluorescein–FITC antibody (Roche, Mannheim, Germany) and the signal was amplified with a rabbit anti-sheep–FITC (DAKO, Denmark). The chromosomes were counterstained with 4,6-diamino-2-phenylindole (DAPI) and the slides were mounted in antifade Vectashield (Vector, Burlingame, CA, USA). Photographs were taken using a Leica microscope equipped with a digital camera, and the images were optimized using Adobe Photoshop CS2.

## Abbreviations

DAPI: 4,6-diamino-2-phenylindole; PCR: Polimerase chain reaction; FISH: Fluorescent in situ hybridization; FITC: Fluorescein isothiocyanate; TRITC: Tetramethyl rhodamine isothiocyanate; SSC: Standard sodium citrate; NOR: Nucleolar organizer region; ITS: Internal transcribed spacer; NTS: non-transcribed spacer; IGS: Intergenic spacer.

## Competing interests

The authors declare that they have no competing interests.

## Authors’ contributions

MSRN carried out the molecular cytogenetic analysis and drafted the manuscript. VL and MJS conceived the work, and participated in its design, drafted and revised the manuscript. All authors analyzed all results and read and approved the final manuscript. VL obtained resources to this work.
